# Supplementary material for: Ubiquitin ligase STUB1 destabilizes IFNγ-receptor complex to suppress tumor IFNγ signaling
Source: Nat Commun. 2022 Apr 8;13:1923. doi: 10.1038/s41467-022-29442-x (PMC8993893; doi:10.1038/s41467-022-29442-x)
Supplement: Supplementary file 1 — Supplementary Information [file 41467_2022_29442_MOESM1_ESM.pdf]

Supplementary Figure 1

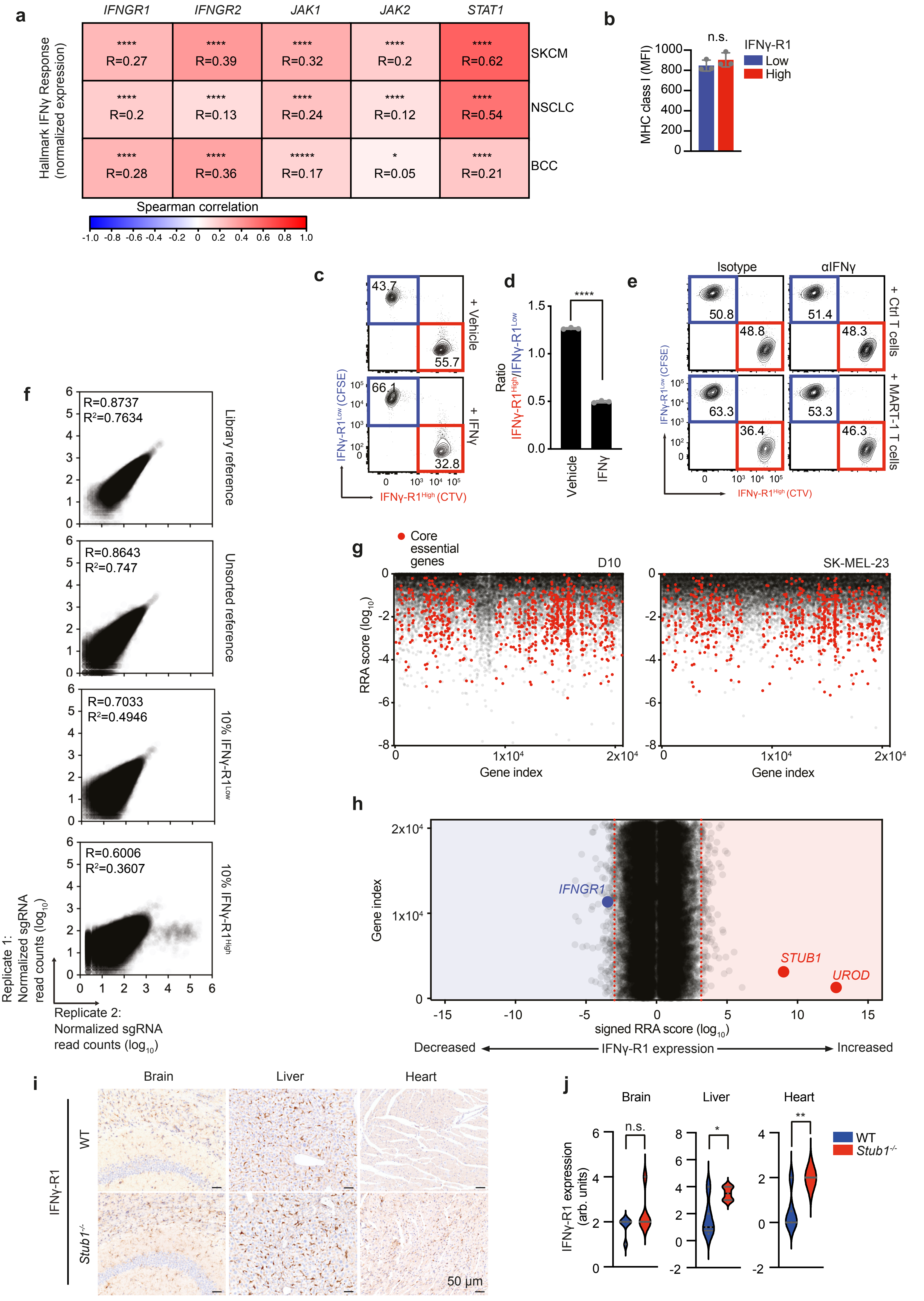

Supplementary Figure Legends

Supplementary Figure 1: Genome-wide CRISPR/Cas9 knockout screen identifies negative regulators of IFNγ-R1 expression to modulate its cell surface abundance.

a, Spearman correlation matrix of *IFNGR1*, *IFNGR2*, *JAK1*, *JAK2* and *STAT1* expression with Hallmark IFNγ response signature in scRNA sequencing data[37–39]. IFNγ-R complex genes present in the Hallmark IFNγ response gene set were removed from the Hallmark IFNγ response gene set prior calculating the correlation. Numbers indicate Spearman correlation.

b, Quantification of MHC class I expression on IFNγ-R1^{High} and IFNγ-R1^{Low}-sorted D10 melanoma cells by flow cytometry from **Figure 1d**.

c, Flow cytometry plot of *in vitro* competition assay of IFNγ-R1^{High} vs. IFNγ-R1^{Low} cells treated with either vehicle or 25 ng/ml IFNγ for five days. Number in quadrants indicates % of parent population.

d, Quantification of the ratio IFNγ-R1^{High} : IFNγ-R1^{Low} in competition assay of (**c**).

e, Flow cytometry plot of the *in vitro* competition assay of IFNγ-R1^{High} vs. IFNγ-R1^{Low} cells co-cultured with either MART-1 or Ctrl T cells. Number in quadrants indicates % of parent population.

f, Spearman correlation plots of log_{10}-transformed normalized read counts of sgRNAs in genome-wide CRISPR-KO screen in D10 melanoma cell line between replicates.

g, Log_{10}-transformed RRA scores of depleted genes comparing library reference sample to unsorted bulk population in D10 (left) and SK-MEL-23 cells (right). Highlighted in red: core essential genes. y-axis: RRA score, x-axis: gene index.

h, Results of screen outlined in (**Figure 1g**) for SK-MEL-23 cells. x-axis: signed log_{10}-transformed signed MAGeCK robust rank aggregation (RRA) score for each gene; y-axis: gene index. Red dotted lines indicate FDR cutoff <0.25 for genes enriched in 10% of cells with the highest (right) or lowest (left) IFNγ-R1 expression.

i, Representative immunohistochemistry images of IFNγ-R1 expression in either wildtype (WT) or *Stub1*-deficient murine brain, liver and heart tissue.

j, Quantification of IFNγ-R1 expression in either WT or *Stub1*-deficient murine brain, liver and heart tissues from **i**.

Mean±SD in (**b**), unpaired t-test for three biological replicates.

Mean±SD in (**d**), ****p<0.0001, unpaired t-test for three biological replicates.

Violin pot in (**j**), **p=0.0047, *p=0.0265 unpaired t-tests for five biological replicates.

Supplementary Figure 2

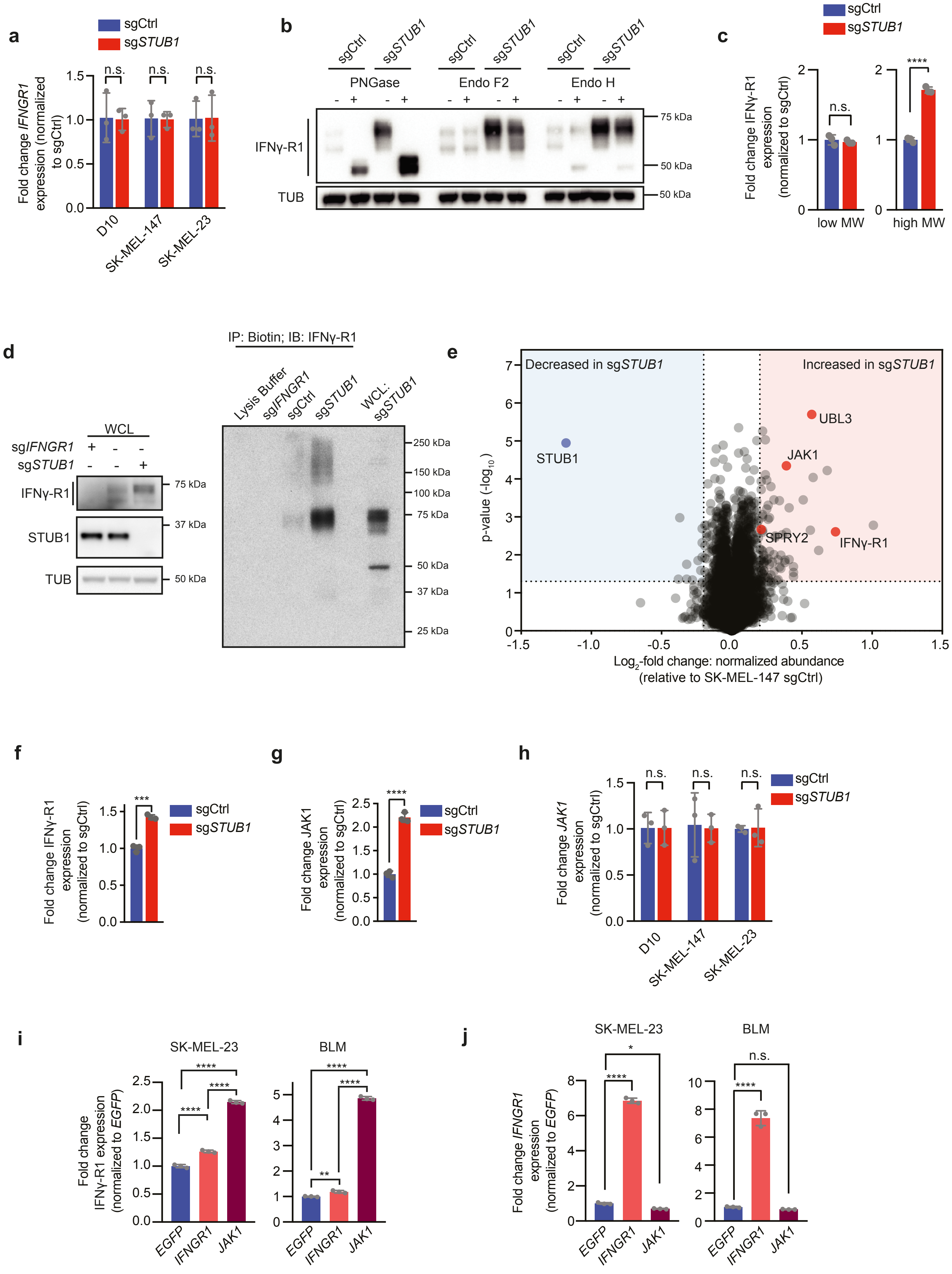

Supplementary Figure 2: STUB1 destabilizes cell surface IFNγ-R1 in JAK1-dependent and JAK1-independent manners.

a, qPCR analysis for *IFNGR1* expression in D10, SK-MEL-147 and SK-MEL-23 cells expressing sgCtrl or sg*STUB1*. *IFNGR1* expression was normalized to sgCtrl-expressing cells using ΔΔCT method.

b, Immunoblot of whole cell lysates (WCL) treated with indicated deglycosylating enzymes. WCL were collected from D10 cells expressing sgCtrl or sg*STUB1* and immunoblotted for IFNγ-R1 and Tubulin. Representative of three biological replicates.

c, Densitometric quantification of low and high molecular weight IFNγ-R1 protein levels (relative to loading control) in D10 cells from immunoblot in (**Figure 2b**).

d, Immunoblot of WCL and immuno-precipitated cell surface proteins using biotin labelling in D10 clone deficient in *IFNGR1*, or D10 cell pool expressing sgCtrl or sg*STUB1*. Immunoprecipitated biotin-labelled proteins were immunoblotted for IFNγ-R1. Right-most lane in the right panel represents 10% of WCL of sg*STUB1*-expressing cells. Representative of three biological replicates.

e, Results of proteomic profiling of SK-MEL-147 cells expressing sgCtrl or sg*STUB1*. Highlighted proteins are differentially regulated in two cell lines (**Figure 2a**).

f, Densitometric quantification of IFNγ-R1 protein levels (relative to loading control) in D10 cells from immunoblot in (**Figure 2b**).

g, same as in (**f**) but for JAK1 protein.

h, qPCR analysis for *JAK1* expression in D10, SK-MEL-147 and SK-MEL-23 cells expressing sgCtrl or sg*STUB1*. *IFNGR1* expression was normalized to sgCtrl-expressing cells using ΔΔCT method.

i, Flow cytometric quantification of IFNγ-R1 expression in SK-MEL-23 and BLM-M cells expressing indicated constructs.

j, qPCR analysis for *IFNGR1* expression in SK-MEL-23 and BLM-M cells expressing indicated constructs. *IFNGR1* expression was normalized to *EGFP*-expressing cells using ΔΔCT method.

Mean±SD in (**a**), unpaired t-tests were performed for each cell line, each three biological replicates.

Mean±SD in (**c**), ****$p<0.0001$, unpaired t-test for three biological replicates.

Mean±SD in (**f**, **g**), unpaired t-test for three biological replicates. ***$p=0.0002$ (**f**), ****$p<0.0001$ (**g**).

Mean±SD in (**h**), multiple t-test for three biological replicates.

Mean±SD in (**i**), **p=0.0093, ****p<0.0001, ordinary one-way ANOVA for three biological replicates with Tukey post hoc testing.

Mean±SD in (**j**), *p=0.0103, ****p<0.0001, ordinary one-way ANOVA for three biological replicates with Dunnett post hoc testing.

Supplementary Figure 3

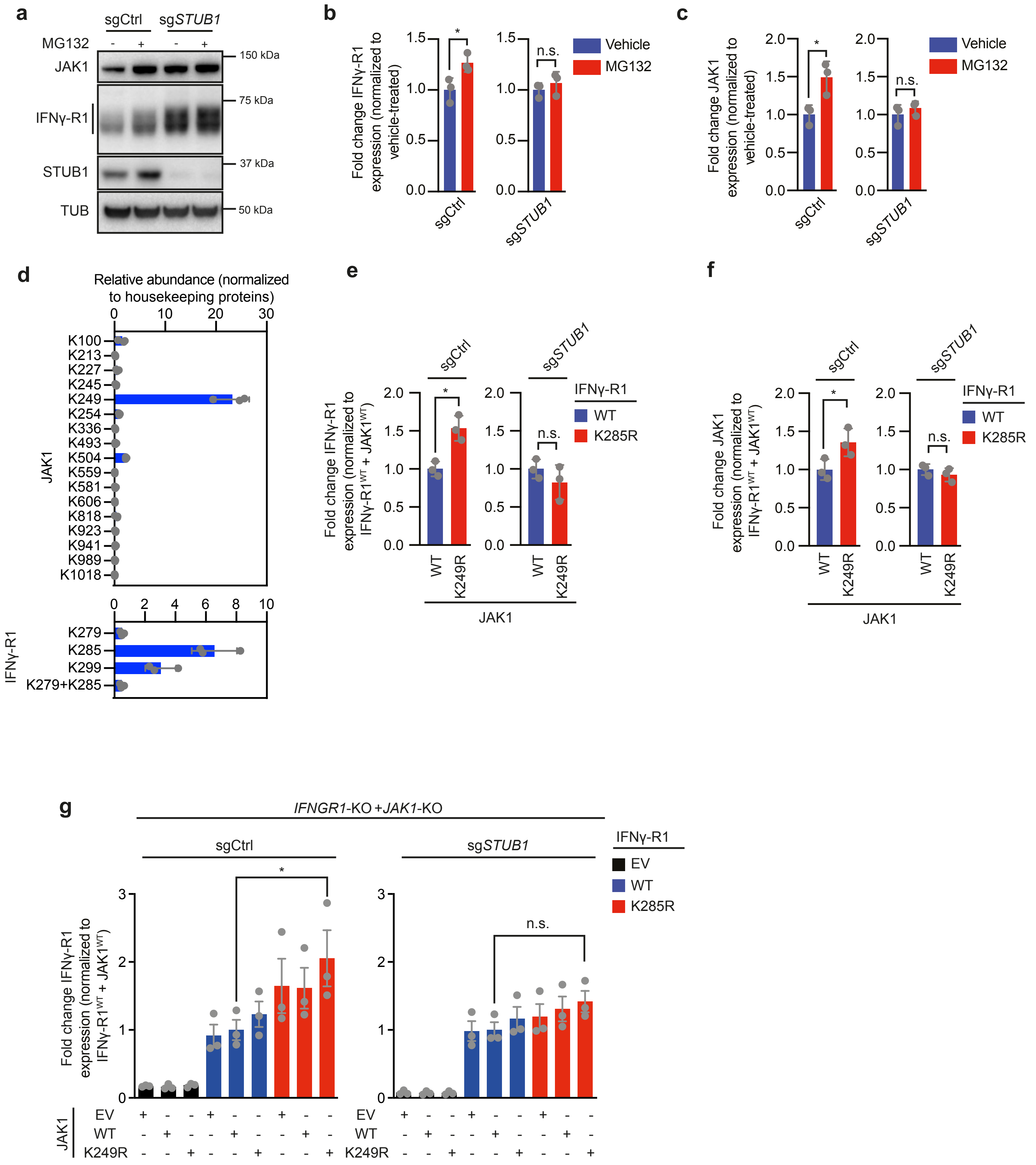

Supplementary Figure 3: STUB1 drives proteasomal degradation of IFNγ receptor complex through IFNγ-R1^{K285} and JAK1^{K249} residues.

a, Immunoblot of SK-MEL-147 cells expressing sgCtrl or sg*STUB1* treated with either vehicle or 10 µM MG132 for four hours. Whole-cell lysates (WCL) were immunoblotted for the indicated proteins (TUB is Tubulin). Representative of three biological replicates.

b, Densitometric quantification of IFNγ-R1 protein levels (relative to loading control and normalized to vehicle-treated group) from (**a**).

c, same as in (**b**) but for JAK1 protein.

d, Relative abundance of ubiquitinated JAK1 and IFNγ-R1 lysine residues in sgCtrl-expressing cells.

e, Densitometric quantification of IFNγ-R1 protein levels on immunoblot in **Figure 3h** (relative to loading control and normalized to IFNγ-R1^{WT} and JAK1^{WT}-expressing cells) in *IFNGR1*-KO + *JAK1*-KO D10 melanoma clones expressing either IFNγ-R1^{WT} and JAK1^{WT} or with IFNγ-R1^{K285R} and JAK1^{K249R}.

f, same as in (**e**) but for JAK1 protein.

g, Flow cytometric quantification of IFNγ-R1 expression in *IFNGR1*-KO + *JAK1*-KO D10 melanoma clones reconstituted with the indicated *IFNGR1* and *JAK1* cDNAs (outlined in **Figure 3d**), shown as fold-change of IFNγ-R1 MFI relative to IFNγ-R1^{WT} + JAK1^{WT}-expressing cells for each respective genotype. EV = empty vector control.

Mean±SD in (**b-g**), ordinary one-way ANOVA for three biological replicates with Tukey post hoc testing. *p=0.0435 (**b**), *p=0.0138 (**c**), *p=0.0156 (**e**), *p=0.0366 (**f**), *p=0.036 (**g**).

Supplementary Figure 4

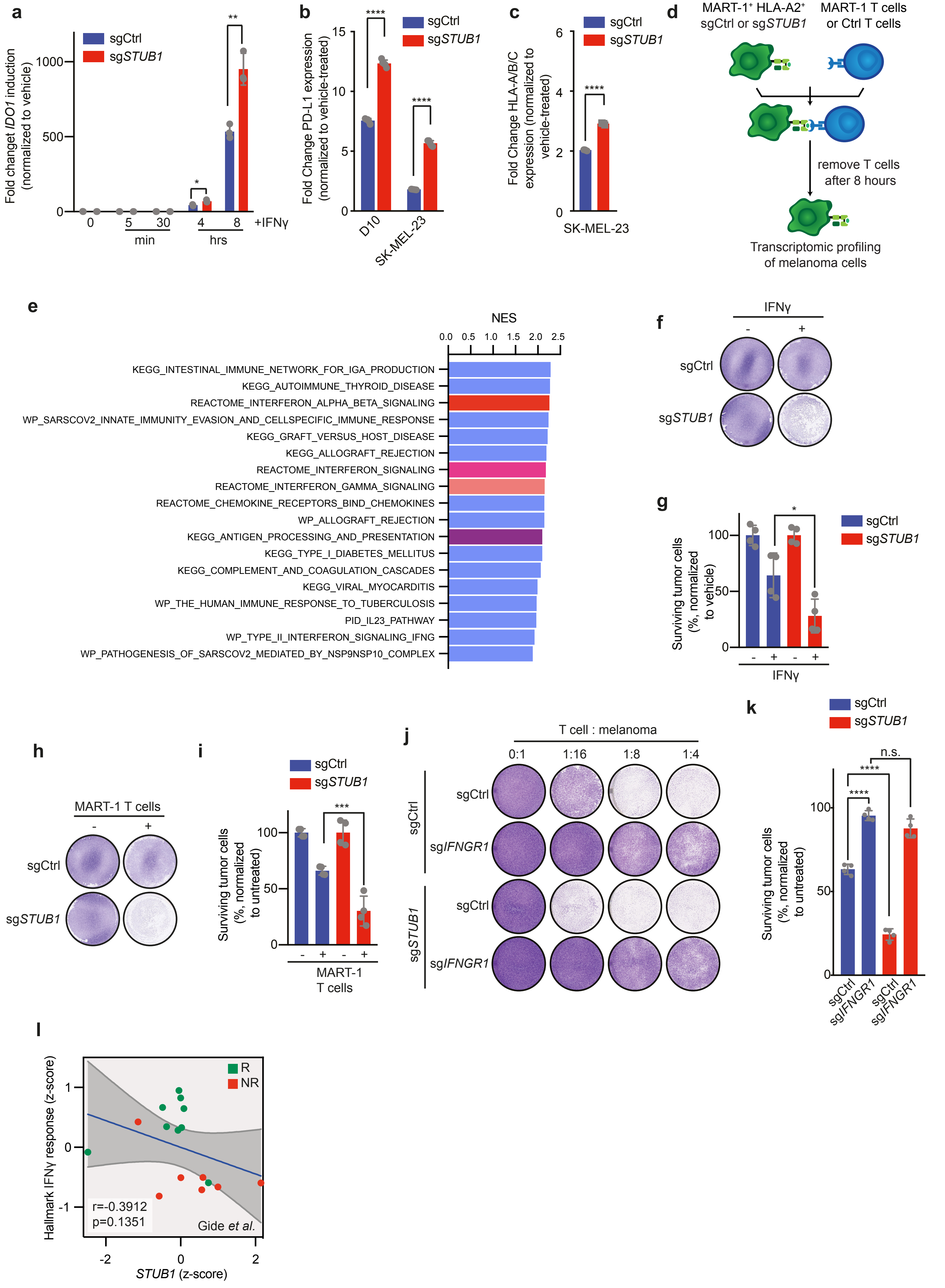

Supplementary Figure 4: *STUB1* inactivation sensitizes melanoma cells to cytotoxic T cells through amplified IFNγ signaling.

a, qPCR analysis for *IDO1* expression in D10 cells expressing sgCtrl or sg*STUB1*, treated with 25 ng/ml IFNγ for the indicated duration.

b, Flow cytometry analysis of IFNγ-induced PD-L1 expression on cells expressing sgCtrl or sg*STUB1* after 24 hours treatment with 5 ng/ml IFNγ for D10 cells and 0.5 ng/ml IFNγ for SK-MEL-23 cells.

c, Flow cytometry analysis of IFNγ-induced HLA-A/B/C expression on SK-MEL-23 cells expressing either sgCtrl or sg*STUB1* after 24 hours treatment with 0.5 ng/ml IFNγ.

d, Schematic outline to transcriptomically profile D10 and SK-MEL-147 cells expressing sgCtrl or sg*STUB1*, after co-cultured with Ctrl or MART-1 T cells for eight hours.

e, Gene set enrichment analysis on RNA sequencing results of cells co-cultured with MART-1 T cells shown in **d**. Depicted are enriched gene sets with FDR<0.05. Highlighted gene sets correspond to gene sets in **Figure 4d.**

f, Colony formation assay of SK-MEL-147 cells expressing sgCtrl or sg*STUB1*, treated with vehicle or 50 ng/ml IFNγ for five days.

g, Quantification of colony formation assay in (**f**).

h, Colony formation assay of SK-MEL-147 cells expressing sgCtrl or sg*STUB1*, treated with no or MART-1 T cells for 24 hours and subsequent culture for four days.

i, Quantification of colony formation assay in (**h**).

j, Colony formation assay of SK-MEL-147 cells expressing indicated sgRNAs, that were co-cultured with no T cell or MART-1 T cells indicated ratios for 24 hours and subsequent culture for four days.

k, Quantification of colony formation assays from (**j**) at a T cell : melanoma cell ratio of 1:16.

l, Spearman correlation of *STUB1* gene expression with the Hallmark IFNγ response gene set expression in patients undergoing anti-PD-1 treatment[71], n=16.

Mean±SD in (**a**), **p=0.0034, *p=0.012, multiple t-tests for three biological replicates.

Mean±SD in (**b**), ****p<0.0001 for SK-MEL-23, ****p<0.0001, unpaired t-test for five biological replicates.

Mean±SD in (**c**), ****p<0.0001, unpaired t-test for five biological replicates.

Mean±SD in (**g**, **i**, **k**), ordinary one-way ANOVA for four biological replicates with Tukey post hoc testing. *p=0.0132 (**g**), ***p=0.0006 (**i**), ****p<0.0001(**k**).

Supplementary Figure 5

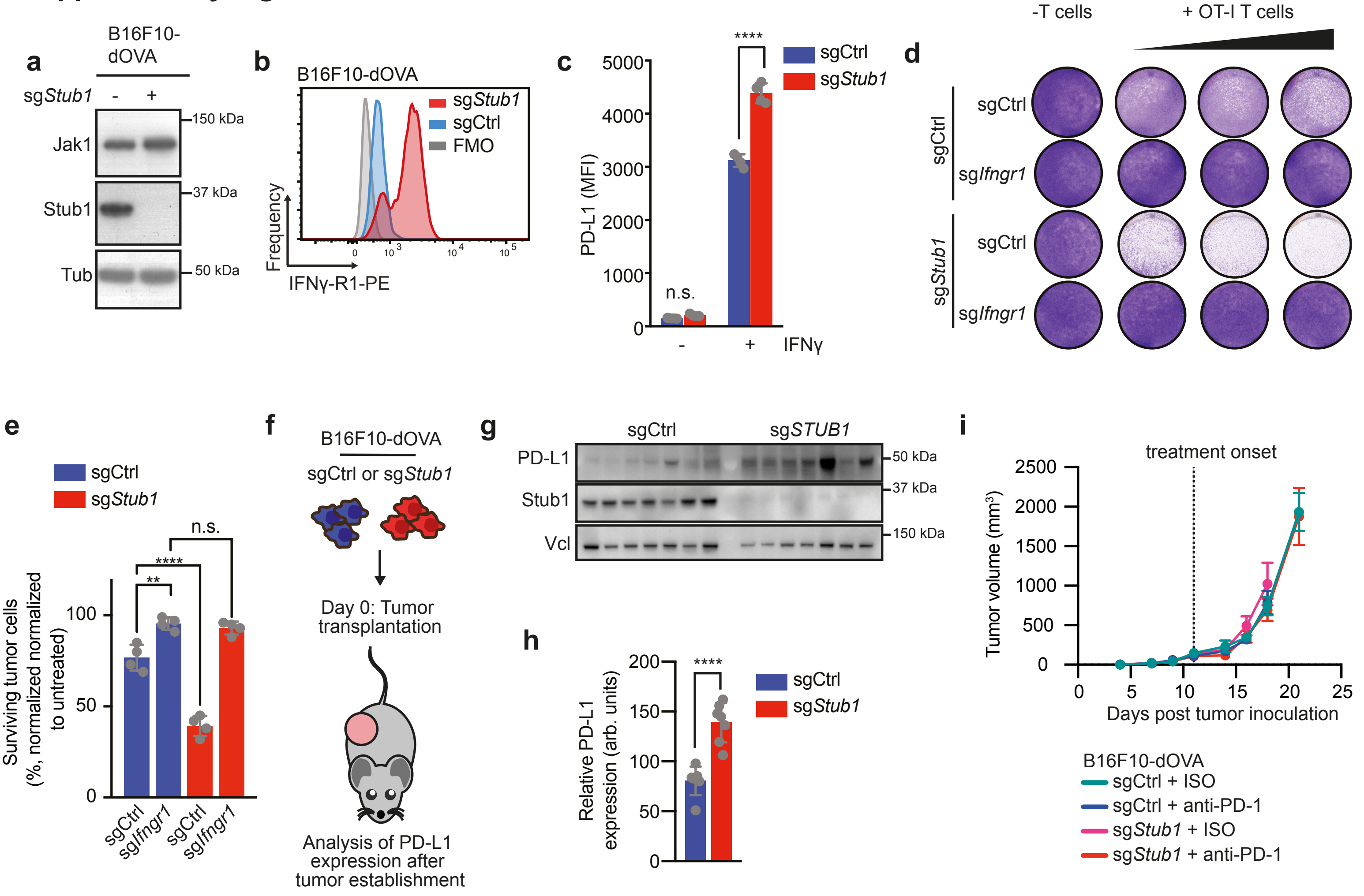

Supplementary Figure 5: STUB1 inactivation enhances IFNγ signaling and increases anti-PD-1 response in heterogeneous tumors with wildtype cells, but not in homogenous STUB1-deficient tumors.

a, Immunoblot of murine melanoma cell line B16F10-dOVA expressing sgCtrl or sg*Stub1*. Whole cell lysates (WCL) were blotted for the indicated proteins (TUB is Tubulin). Representative of three biological replicates.

b, Flow cytometry quantification of IFNγ-R1 expression in B16F10-dOVA expressing sgCtrl (blue) or sg*Stub1* (red). FMO (grey) = Fluorescence minus one, PE=Phycoerythrin.

c, Flow cytometry analysis of IFNγ-induced PD-L1 expression in B16F10-dOVA cells expressing sgCtrl or sg*Stub1*. Cells were treated with 12 ng/ml murine IFNγ for 24 hours.

d, Colony formation assay of B16F10-dOVA melanoma cells expressing the indicated sgRNAs and co-cultured with no T cells or OT-I T cells at T cell : melanoma cell ratios 1:1, 2:1 and 4:1 (left to right).

e, Quantification from (**d**) at a T cell : melanoma cell ratio of 4:1.

f, Experimental outline to assess PD-L1 expression on either sgCtrl or sg*Stub1*-expressing B16F10-dOVA tumors *in vivo*.

g, Immunoblot of B16F10-dOVA *in vivo* tumor samples expressing sgCtrl or sg*Stub1* (outlined in **f**) for the indicated proteins (Vcl is vinculin). n=7 tumors per group.

h, Densitometric quantification of PD-L1 protein levels (relative to loading control) of tumor samples from immunoblot shown in (**g**).

i, *In vivo* tumor volumes of sgCtrl- or sg*Stub1*-expressing B16F10-dOVA melanoma tumors in immune-competent mice treated with isotype control antibody (ISO) or murine anti-PD-1. Dashed line marks start of a twice-weekly anti-PD-1 treatment.

Mean±SD in (**c**, **e**), ordinary one-way ANOVA for four biological replicates with Tukey post hoc testing. ****p $p<0.0001$ (**c**), **$p=0.0012$, ****$p<0.0001$ (**e**).

Mean±SD in (**h**), **** $p<0.0001$, unpaired two-tailed t-test, n=7 tumors per group.

Mean±SEM in (**i**), n.s. multiple Mann-Whitney tests for n=10 tumors per group, except B16F10-oOVA sg*Stub1* + anti-PD-1 n=9.

Supplementary Figure 6

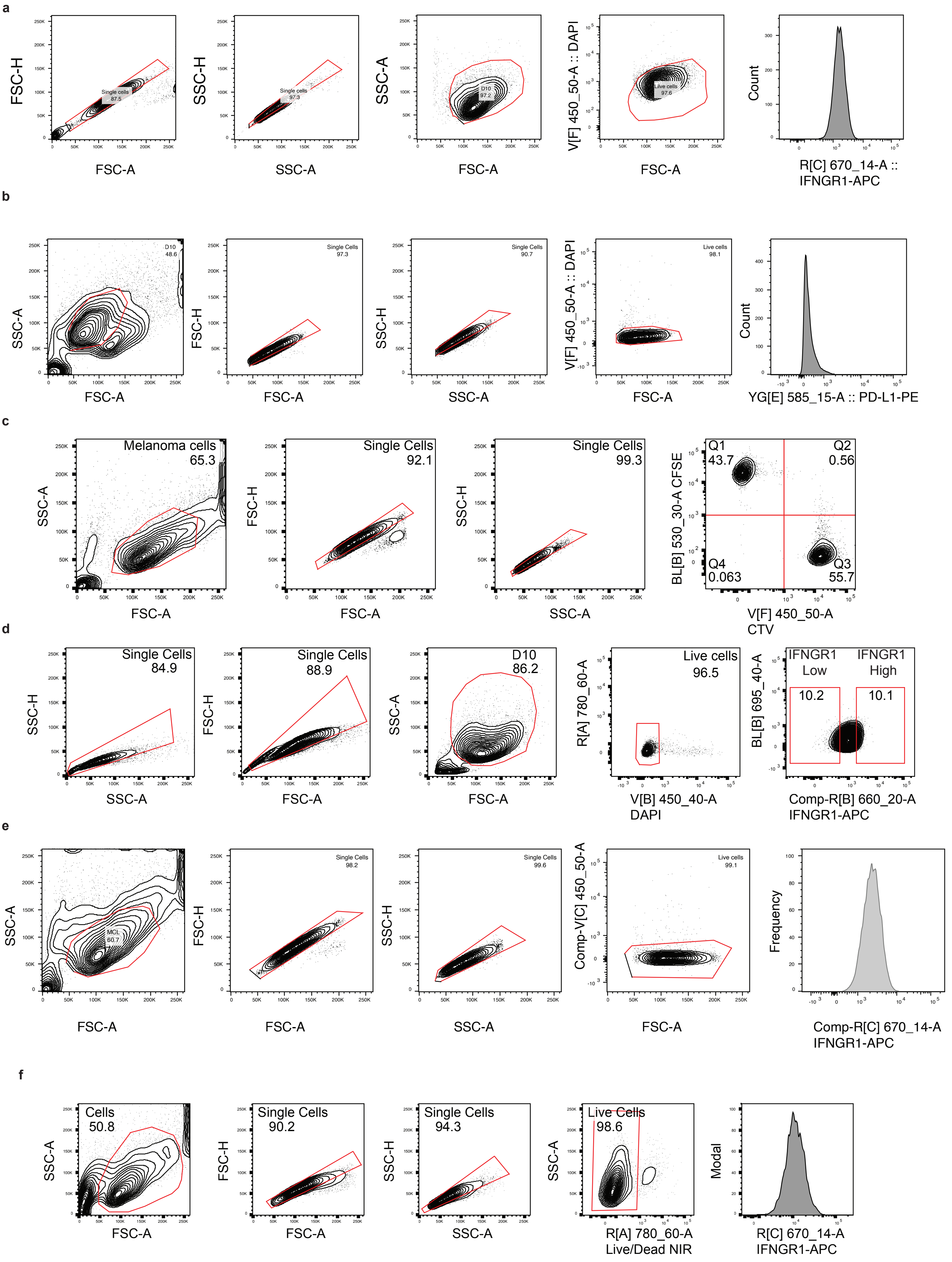

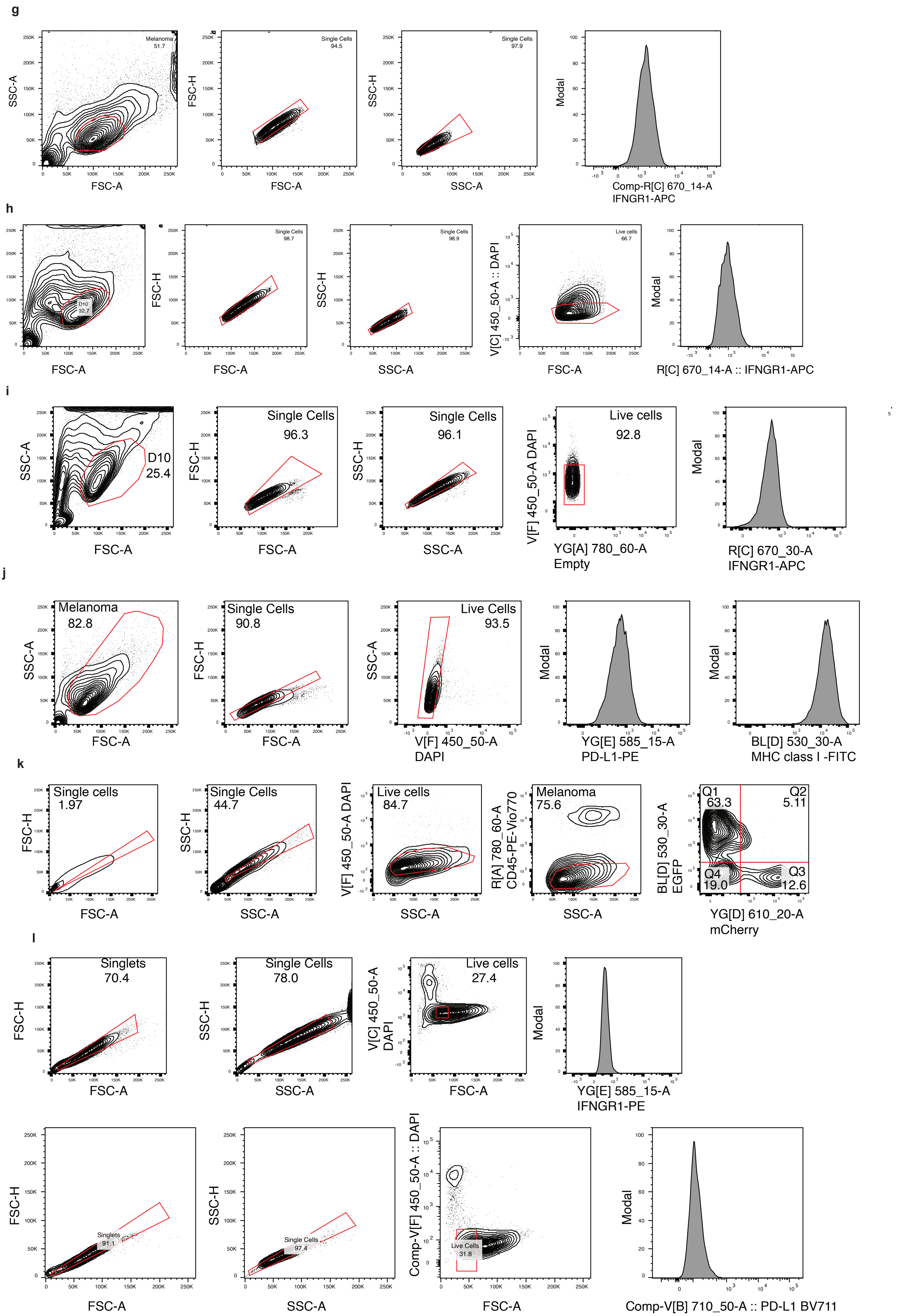
g
Melanoma
51.7
SSC-A
FSC-A
Single Cells
94.5
FSC-H
FSC-A
Single Cells
97.9
SSC-H
SSC-A
Modal
Comp-R[C] 670_14-A
IFNGR1-APC
h
SSC-A
D10
32.7
FSC-A
Single Cells
98.7
FSC-H
FSC-A
Single Cells
98.9
SSC-H
SSC-A
Live cells
66.7
V[C] 450_50-A :: DAPI
FSC-A
Modal
R[C] 670_14-A :: IFNGR1-APC
i
SSC-A
D10
25.4
FSC-A
Single Cells
96.3
FSC-H
FSC-A
Single Cells
96.1
SSC-H
SSC-A
Live cells
92.8
V[F] 450_50-A DAPI
YG[A] 780_60-A
Empty
Modal
R[C] 670_30-A
IFNGR1-APC
j
Melanoma
82.8
SSC-A
FSC-A
Single Cells
90.8
FSC-H
FSC-A
Live Cells
93.5
SSC-A
V[F] 450_50-A
DAPI
Modal
YG[E] 585_15-A
PD-L1-PE
Modal
BL[D] 530_30-A
MHC class I -FITC
k
Single cells
1.97
FSC-H
FSC-A
Single Cells
44.7
SSC-H
SSC-A
Live cells
84.7
V[F] 450_50-A DAPI
SSC-A
Melanoma
75.6
R[A] 780_60-A
CD45-PE-Vio770
SSC-A
Q1
63.3
Q2
5.11
Q4
19.0
Q3
12.6
BL[D] 530_30-A
EGFP
YG[D] 610_20-A
mCherry
l
Singlets
70.4
FSC-H
FSC-A
Single Cells
78.0
SSC-H
SSC-A
Live cells
27.4
V[C] 450_50-A
DAPI
FSC-A
Modal
YG[E] 585_15-A
IFNGR1-PE
m
FSC-H
Singlets
91.1
FSC-A
SSC-H
Single Cells
97.4
SSC-A
Comp-V[F] 450_50-A :: DAPI
Live Cells
31.8
FSC-A
Modal
Comp-V[B] 710_50-A :: PD-L1 BV711

Supplementary Figure 6: Gating Strategy for FACS plots

a, Gating strategy corresponding to Figure 1d.

b, Gating strategy for Figure 1e and Supplementary Figure 1b.

c, Gating strategy for Figure 1f, Supplementary Figure 1c and e.

d, Gating strategy for Figure 1g.

e, Gating strategy for Figure 1i.

f, Gating strategy for Figure 1j, k.

g, Gating strategy for Figure 2d and Supplementary Figure 2i.

h, Gating strategy for Figure 2j.

i, Gating strategy for Figure 3i and Supplementary Figure 3g.

j, Gating strategy for Supplementary Figure 4b, c.

k, Gating strategy for Figure 5d, e.

l, Gating strategy for Supplementary Figure 5b, c.

m, Gating strategy for Supplementary Figure 5c.
