# Supplementary material for: Ubiquitin ligase STUB1 destabilizes IFNγ-receptor complex to suppress tumor IFNγ signaling
Source: Nat Commun. 2022 Apr 8;13:1923. doi: 10.1038/s41467-022-29442-x (PMC8993893; doi:10.1038/s41467-022-29442-x)
Supplement: Supplementary file 2 — Reporting Summary [file 41467_2022_29442_MOESM2_ESM.pdf]

nature research

Corresponding author(s): Daniel Peeper

Last updated by author(s): Feb 7, 2022

Reporting Summary

Nature Research wishes to improve the reproducibility of the work that we publish. This form provides structure for consistency and transparency in reporting. For further information on Nature Research policies, see Authors & Referees and the Editorial Policy Checklist .

Statistics

For all statistical analyses, confirm that the following items are present in the figure legend, table legend, main text, or Methods section.

n/a	Confirmed	
☐	☒	The exact sample size (*n*) for each experimental group/condition, given as a discrete number and unit of measurement
☐	☒	A statement on whether measurements were taken from distinct samples or whether the same sample was measured repeatedly
☐	☒	The statistical test(s) used AND whether they are one- or two-sided<br>*Only common tests should be described solely by name; describe more complex techniques in the Methods section.*
☐	☒	A description of all covariates tested
☐	☒	A description of any assumptions or corrections, such as tests of normality and adjustment for multiple comparisons
☐	☒	A full description of the statistical parameters including central tendency (e.g. means) or other basic estimates (e.g. regression coefficient) AND variation (e.g. standard deviation) or associated estimates of uncertainty (e.g. confidence intervals)
☐	☒	For null hypothesis testing, the test statistic (e.g. *F*, *t*, *r*) with confidence intervals, effect sizes, degrees of freedom and *P* value noted<br>*Give P values as exact values whenever suitable.*
☒	☐	For Bayesian analysis, information on the choice of priors and Markov chain Monte Carlo settings
☒	☐	For hierarchical and complex designs, identification of the appropriate level for tests and full reporting of outcomes
☐	☒	Estimates of effect sizes (e.g. Cohen's *d*, Pearson's *r*), indicating how they were calculated

Our web collection on statistics for biologists contains articles on many of the points above.

Software and code

Policy information about availability of computer code

Data collection	STAR(2.6.0c), HTSeq-count (v0.10.0), DESeq2 (v1.24.0), FACSDiva (v8.0)
Data analysis	Graphpad Prism (v8.0.0 131), FlowJo (v10.6.0), GSEA (v4.0.3), MAGeCK (v0.5.7), R Studio (v1.1.463) with packages as indicated, Tophat v2.1, Perseus software (v1.5.6.0),), Proteome Discoverer 2.2.

For manuscripts utilizing custom algorithms or software that are central to the research but not yet described in published literature, software must be made available to editors/reviewers. We strongly encourage code deposition in a community repository (e.g. GitHub). See the Nature Research guidelines for submitting code & software for further information.

Data

Policy information about availability of data

All manuscripts must include a data availability statement. This statement should provide the following information, where applicable:
- Accession codes, unique identifiers, or web links for publicly available datasets
- A list of figures that have associated raw data
- A description of any restrictions on data availability

RNA sequencing data in this manuscript is available at GSE154040.
Proteomics data have been deposited on Proteome Exchange with the accession nr PXD030580.
The sequencing data of the genome-wide CRISPR/Cas9 screens in D10 and SK-MEL-23 melanoma cells is provided as part of the source data.

Field-specific reporting

Please select the one below that is the best fit for your research. If you are not sure, read the appropriate sections before making your selection.

Life sciences study design

All studies must disclose on these points even when the disclosure is negative.

Sample size	Each experiment was performed using at least three independent biological replicates. For the in vivo experiment, an a priori power analysis was performed using G*Power 2 (v3.1.9.2) with the effect size being determined by a preliminary pilot experiment.
Data exclusions	No data were excluded.
Replication	All findings were successfully replicated in at least three independent experiments.
Randomization	For the in vivo experiments, randomization occurred at time of treatment by an independently operating technician. Randomization does not applicable to the reported in vitro experiments, since either different treatments or genotypes were compared.
Blinding	For in vivo experiments researchers were blinded to treatment groups. Additionally, tumor volume measurements were performed by independent mouse technicians and not by primary researchers themselves.

Reporting for specific materials, systems and methods

We require information from authors about some types of materials, experimental systems and methods used in many studies. Here, indicate whether each material, system or method listed is relevant to your study. If you are not sure if a list item applies to your research, read the appropriate section before selecting a response.

Materials & experimental systems

n/a	Involved in the study	
☐	☒	Antibodies
☐	☒	Eukaryotic cell lines
☒	☐	Palaeontology
☐	☒	Animals and other organisms
☒	☐	Human research participants
☒	☐	Clinical data

Methods

n/a	Involved in the study	
☒	☐	ChIP-seq
☐	☒	Flow cytometry
☒	☐	MRI-based neuroimaging

Antibodies

Antibodies used

Antibodies for flow cytometry were used at a dilution of 1:100 if not stated otherwise.

Flow Cytometry: anti-PD-L1 (eBioscience, 12-5983-42), anti-HLA-A/B/C (RND Systems, FAB7098G), anti-CD119/IFNGR1 (Miltenyi Biotech, 130-099-921), anti-CD45-APC (Miltenyi, 130-102-544), anti-mouse-CD279 (563369, BD Bioscience).

T cell activation: 5µg/ml anti-CD3 (eBioscience, 16-0037-85) and 5µg/ml anti-CD28 (eBioscience, 16-0289-85)

Immuno-blot: anti-IFNGR1 (Santa Cruz Biotechnology, sc-28363, dilution: 1:200), anti-JAK1 (clone D1T6W, Cell Signaling Technology, 50996, dilution: 1:1000), anti-STUB1/CHIP (C3B6, Cell Signaling Technology, 2080, dilution: 1:1000), anti-Tubulin (DM1A, Sigma Aldrich, T9026, dilution: 1:1000), anti-STAT1 (D1K9Y, Cell Signaling Technology, 12994, dilution: 1:1000), anti-STAT1-Tyr701 (58D6, Cell Signaling Technology, 9167, dilution: 1:1000), anti-FLAG (F3040-.2MG ,Sigma Aldrich, dilution: 1:1000), Anti-V5 (R960-25, Invitrogen, dilution: 1:1000), anti-Myc (9E10 clone, in house, dilution: 1:1000).

In vivo experiments: 100µg per injection of anti-mouse-PD-1 clone RMP1-14 (P372, Leinco Technologies).

Validation

All antibodies were validated for their applications by the manufacturer or by ourselves using genetic knockouts.

anti-PD-L1 (eBioscience, 12-5983-42):https://www.thermofisher.com/order/genome-database/dataSheetPdf?producttype=antibody&productsubtype=antibody_primary&productId=12-5983-42&version=205

anti-HLA-A/B/C (RND Systems, FAB7098G): https://resources.rndsystems.com/pdfs/datasheets/fab7098g.pdf?v=20220204&_ga=2.255770568.1370089504.1643979602-1227718772.1632325190

anti-CD119/IFNGR1 (Miltenyi Biotech, 130-099-921): https://www.citeab.com/antibodies/2092167-130-099-921-cd119-apc-human

anti-CD45-APC (Miltenyi, 130-102-544): https://www.citeab.com/antibodies/2090199-130-102-544-cd45-apc-mouse

anti-mouse-CD279 (563369, BD Bioscience): https://www.bdbiosciences.com/content/dam/bdb/products/global/reagents/flow-cytometry-reagents/research-reagents/single-color-antibodies-ruo/563369_base/pdf/563369.pdf

anti-CD3 (eBioscience, 16-0037-85): https://www.thermofisher.com/order/genome-database/dataSheetPdf?producttype=antibody&productsubtype=antibody_primary&productId=16-0037-85&version=205

anti-CD28 (eBioscience, 16-0289-85): https://www.thermofisher.com/order/genome-database/dataSheetPdf?producttype=antibody&productsubtype=antibody_primary&productId=16-0289-85&version=205

anti-IFNGR1 (Santa Cruz Biotechnology, sc-28363): https://datasheets.scbt.com/sc-28363.pdf

anti-JAK1 (clone D1T6W): https://www.cellsignal.com/datasheet.jsp?productId=50996&images=1

anti-STUB1/CHIP (C3B6): https://www.cellsignal.com/datasheet.jsp?productId=2080&images=1

anti-Tubulin (DM1A): https://www.thermofisher.com/order/genome-database/dataSheetPdf?producttype=antibody&productsubtype=antibody_primary&productId=14-4502-82&version=205

anti-STAT1 (D1K9Y): https://www.cellsignal.com/datasheet.jsp?productId=14994&images=1
anti-STAT1-Tyr701 (58D6): https://www.cellsignal.com/datasheet.jsp?productId=9167&images=1
anti-FLAG (F3040-.2MG): https://www.sigmaaldrich.com/specification-sheets/407/239/F3040-5MG_________SIGMA____.pdf
Anti-V5 (R960-25): https://www.thermofisher.com/order/genome-database/dataSheetPdf?producttype=antibody&productsubtype=antibody_primary&productId=R960-25&version=205
anti-Myc (9E10): In house production.
anti-mouse-PD-1 clone RMP1-14 (P372, Leinco Technologies): https://www.leinco.com/p/anti-mouse-pd-1-cd279-purified-3/

Eukaryotic cell lines

Policy information about cell lines

Cell line source(s)	The human D10 (female), SK-MEL-23 (female), SK-MEL147 (female), A375 (female), SK-MEL-28 (male), BLM-M (male), 451Lu (male), A101D (male), LCLC-103H (male), HCC-4006 (male), RKO (unspecified), 8505C (female) and HEK293T (female) cell lines were obtained from the internal Peeper laboratory stock, as was the murine B16F10-OVA (male) cell line.
Authentication	Cell lines were authenticated using the STR profiling kit by Promega (B9510).
Mycoplasma contamination	All cell lines were tested monthly by PCR to be negative for mycoplasma infection. If cell lines were found to be infected, they were immediately discarded.
Commonly misidentified lines (See ICLAC register)	No commonly misidentified cell lines were used in this study

Animals and other organisms

Policy information about studies involving animals; ARRIVE guidelines recommended for reporting animal research

Laboratory animals	Mus musculus, C57BL/6 (Janvier) and NSG-B2m (Jackson Laboratories), male, age 8-12 weeks.
Wild animals	This study did not involve wild animals
Field-collected samples	This study did not involve field-collected samples.
Ethics oversight	All animal studies were approved by the animal ethics committee of the Netherlands Cancer Institute (NKI) and performed in accordance with ethical and procedural guidelines established by the NKI and Dutch legislation.

Note that full information on the approval of the study protocol must also be provided in the manuscript.

Flow Cytometry

Plots

Confirm that:

- [x] The axis labels state the marker and fluorochrome used (e.g. CD4-FITC).
- [x] The axis scales are clearly visible. Include numbers along axes only for bottom left plot of group (a 'group' is an analysis of identical markers).
- [x] All plots are contour plots with outliers or pseudocolor plots.
- [x] A numerical value for number of cells or percentage (with statistics) is provided.

Methodology

Sample preparation	Cell lines were washed with PBS and single cell suspensions were generated using trypsin digestion. Cells were subsequently washed with 0.1% BSA/PBS and stained with the respective antibodies (diluted in 0.1% BSA/PBS) for 30 minutes on ice. Cells were washed twice with 0.1% BSA/PBS and DAPI was added to each sample to determine the percentage of dead cells. Transplanted tumors were harvested dissociated into small pieces using scissors. To generate a single cell suspension, each tumor was digested using collagenase IV and subsequently forced through 70 µm cell strainers. The resulting single cell suspension was prepared for flow cytometry as stated above.
Instrument	Samples were analyzed using BD LSR Fortessa Cell analyzer, cells were sorted on the BD FACSAria Illu
Software	The FACSDiva (V8) software was used to collect flow cytometry data. FlowJo software (V10) was used to analyze flow cytometry data.
Cell population abundance	For the sort-based screen, we sorted 10% of cells with the highest and 10% of cells with the lowest expression levels of IFNGR1 from the viable (DAPI-negative) IFNGR1-APC-positive cells.

Gating strategy

The cell population was identified using FSC and SSC. Cell doublets were excluded using SSC-A vs SSC-H and subsequent FSC-A vs FSC-H gatings. Live cells were determined by their lack of DAPI-positivity. Boundaries for positive and negative populations were determined using either unstained control samples or fluorescence minus one (FMO) control samples.

☒ Tick this box to confirm that a figure exemplifying the gating strategy is provided in the Supplementary Information.
