# Supplementary material for: Ubiquitin ligase STUB1 destabilizes IFNγ-receptor complex to suppress tumor IFNγ signaling
Source: Nat Commun. 2022 Apr 8;13:1923. doi: 10.1038/s41467-022-29442-x (PMC8993893; doi:10.1038/s41467-022-29442-x)
Supplement: Supplementary file 4 — Description of Additional Supplementary Files [file 41467_2022_29442_MOESM4_ESM.pdf]

Title: Supplementary Data 1:
Description: Read count table for CRISPR/Cas9 knockout screen in D10 cells.

Title: Supplementary Data 2:
Description: Read count table for CRISPR/Cas9 knockout screen in SK-MEL-23 cells.

Title: Supplementary Data 3:
Description:DESeq results of T cell response in STUB1-KO cells in D10 and SK-MEL-147 cells.

Title: Supplementary Data 4:
Description: Gene set enrichment analysis summary table.
