# Supplementary material for: Ubiquitin ligase STUB1 destabilizes IFNγ-receptor complex to suppress tumor IFNγ signaling
Source: Nat Commun. 2022 Apr 8;13:1923. doi: 10.1038/s41467-022-29442-x (PMC8993893; doi:10.1038/s41467-022-29442-x)

Fig. 2b

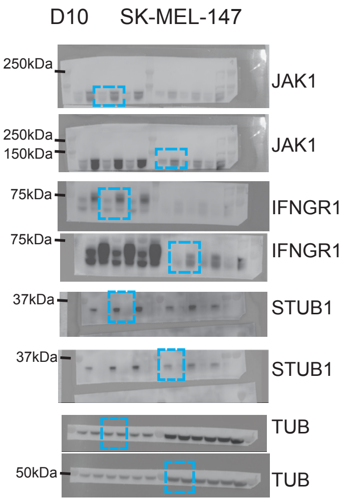

Fig. 2c

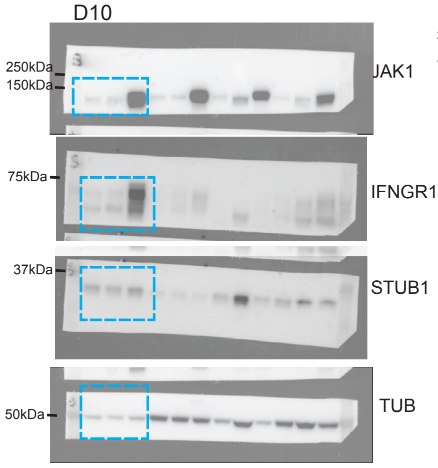

Fig. 2f

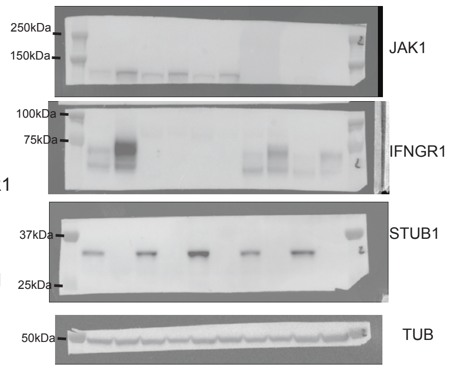

Fig. 2i

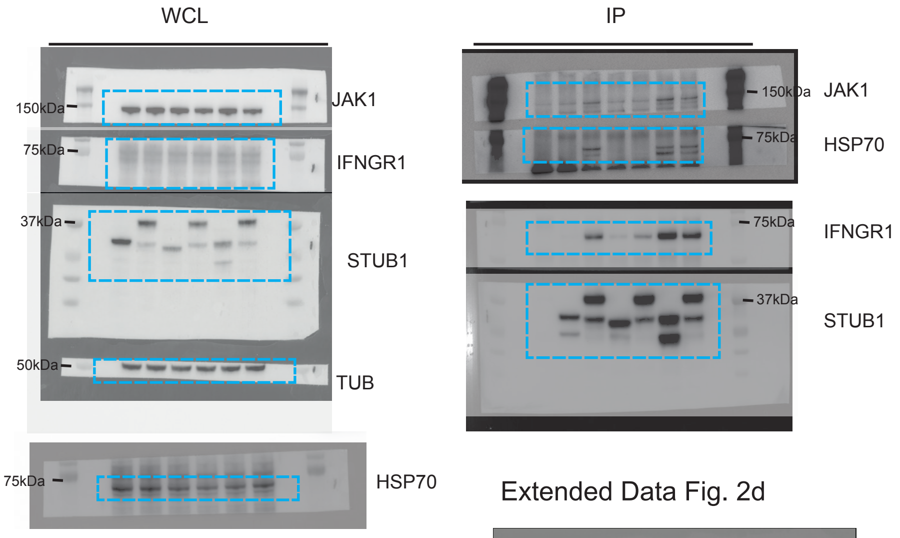

Fig. 2k

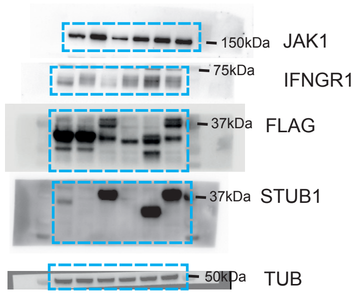

Extended Data Fig. 2d

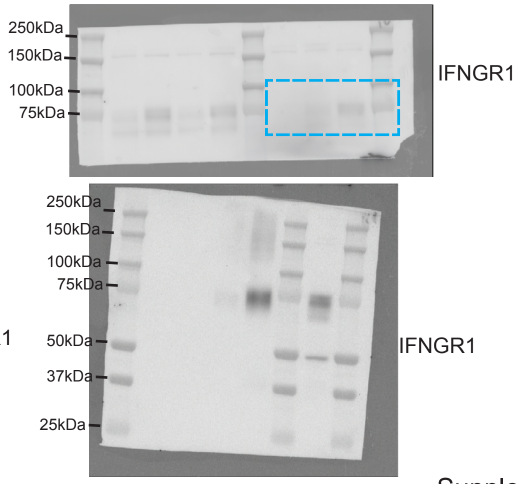

Fig. 3a

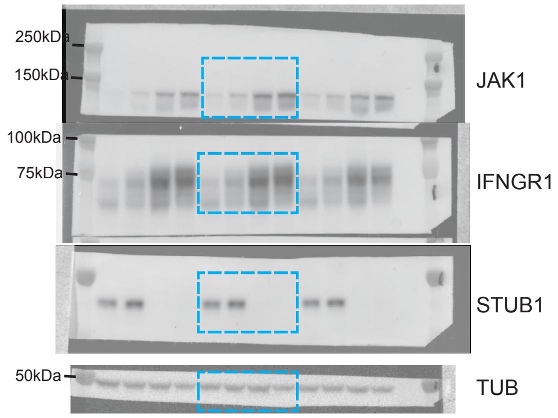

Extended Data Fig. 2b

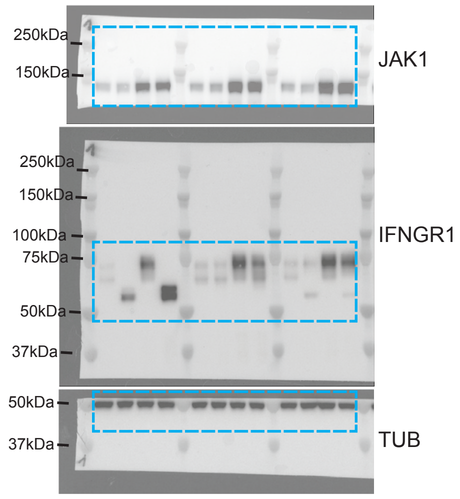

Supplementary Fig. 3a

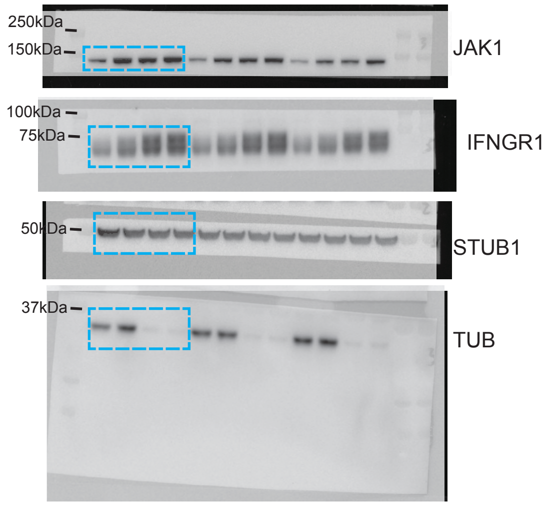

Fig. 3e

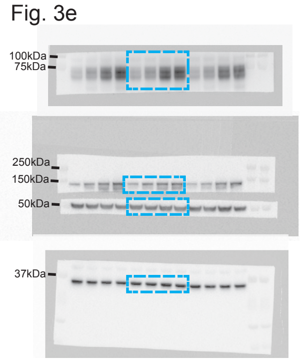

Fig. 3h

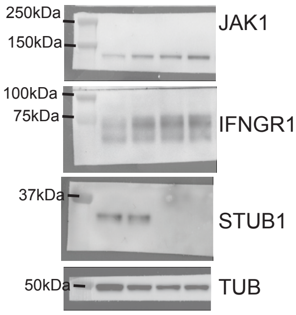

Fig. 4a

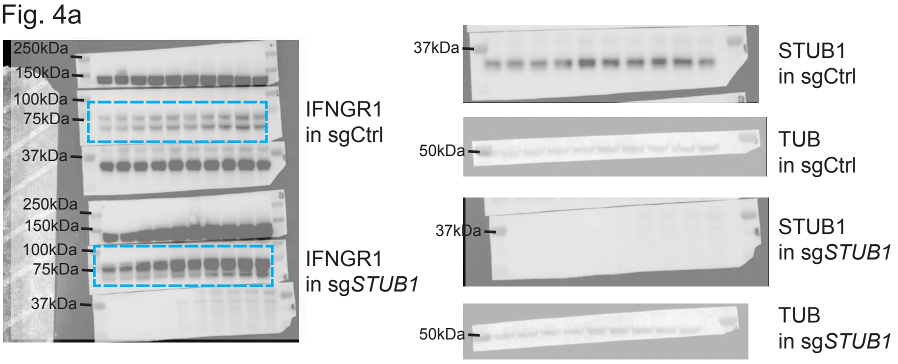

Fig. 4b

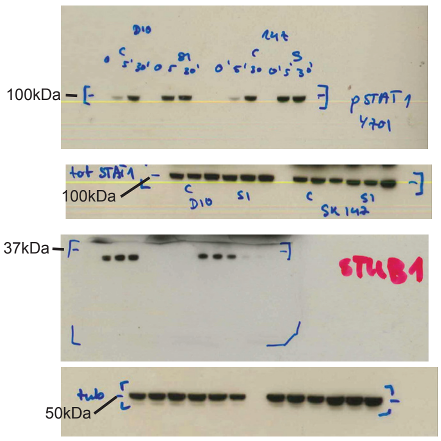

Supplementary Fig. 5a

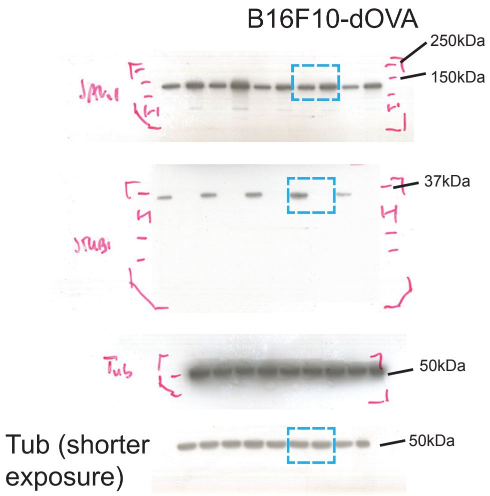

Supplementary Fig. 5g

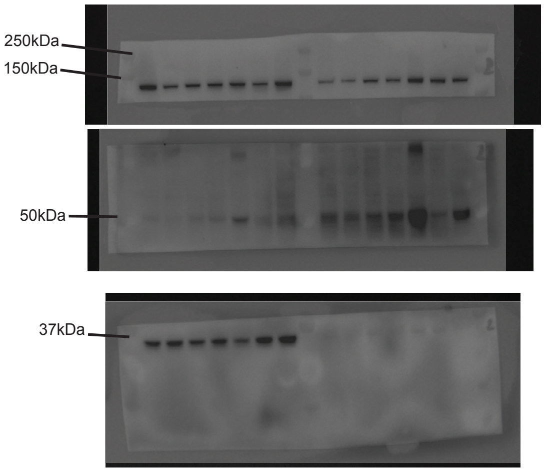

Supplement: Supplementary file 9 — Source Data File [file 41467_2022_29442_MOESM9_ESM.zip › Source Data File_Western blot overlays.pdf]
